# Development of rapid nucleic acid testing techniques for common respiratory infectious diseases in the Chinese population

**DOI:** 10.3389/fchem.2024.1381738

**Published:** 2024-04-17

**Authors:** Shenshen Zhi, Wenyan Wu, Yan Ding, Yuanyuan Zhang, Liyan Pan, Guo Liu, Wei Li

**Affiliations:** ^1^ Department of Blood Transfusion, Chongqing Emergency Medical Center, School of Medicine, Chongqing University Central Hospital, Chongqing University, Chongqing, China; ^2^ Clinical Laboratory, Chongqing Emergency Medical Center, School of Medicine, Chongqing University Central Hospital, Chongqing University, Chongqing, China; ^3^ Zeal Dental, Chongqing, China

**Keywords:** rapid testing, nucleic acid, respiratory infections, fluorescent quantitative PCR, public health

## Abstract

**Background:** Most respiratory viruses can cause serious lower respiratory diseases at any age. Therefore, timely and accurate identification of respiratory viruses has become even more important. This study focused on the development of rapid nucleic acid testing techniques for common respiratory infectious diseases in the Chinese population.

**Methods:** Multiplex fluorescent quantitative polymerase chain reaction (PCR) assays were developed and validated for the detection of respiratory pathogens including the novel coronavirus (SARS-CoV-2), influenza A virus (FluA), parainfluenza virus (PIV), and respiratory syncytial virus (RSV).

**Results:** The assays demonstrated high specificity and sensitivity, allowing for the simultaneous detection of multiple pathogens in a single reaction. These techniques offer a rapid and reliable method for screening, diagnosis, and monitoring of respiratory pathogens.

**Conclusion:** The implementation of these techniques might contribute to effective control and prevention measures, leading to improved patient care and public health outcomes in China. Further research and validation are needed to optimize and expand the application of these techniques to a wider range of respiratory pathogens and to enhance their utility in clinical and public health settings.

## 1 Introduction

**FIGURE 1:**
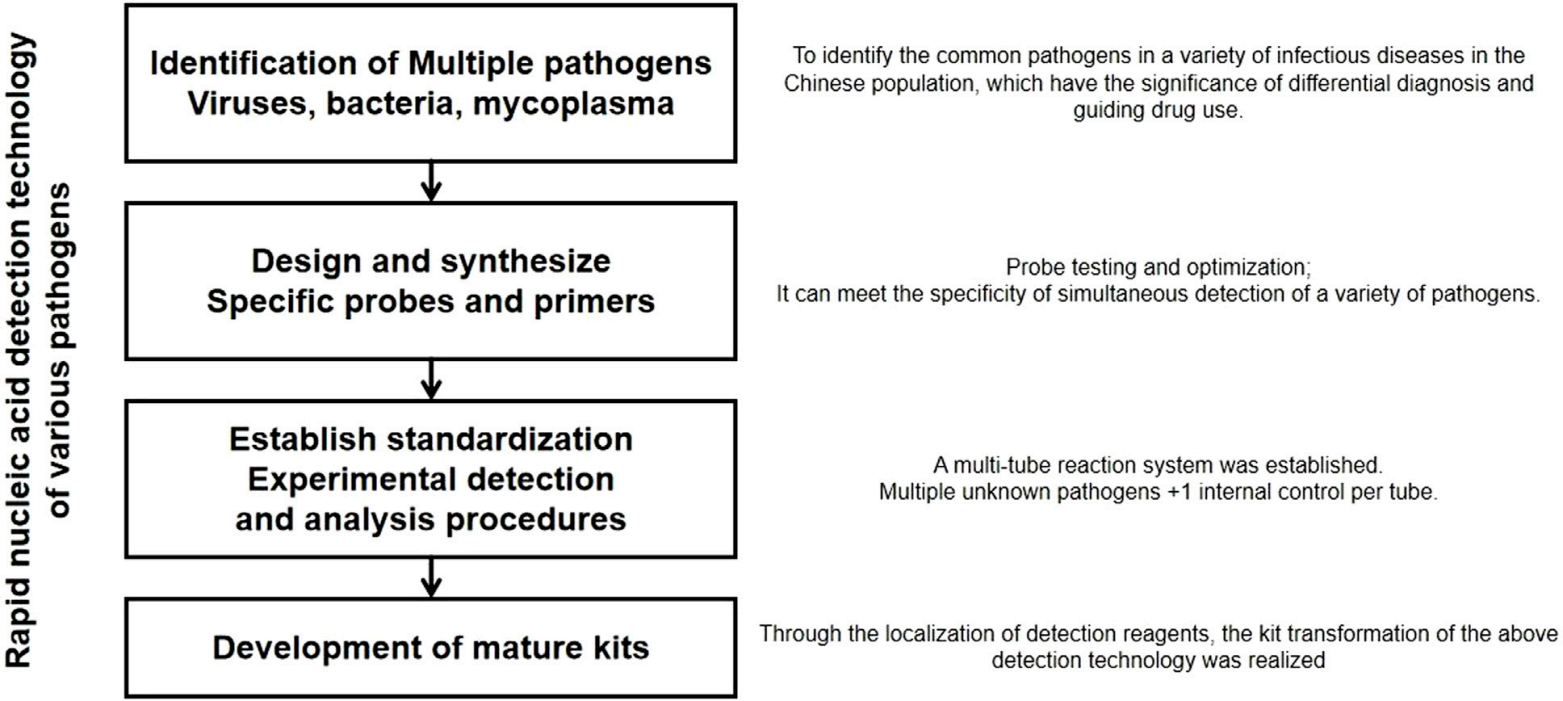
Experimental scheme.

The novel coronavirus discovered in 2019 (COVID-19) has caused numerous cases of infection and death worldwide (Agostoni et al., 2020; Clinical, 2021; Petti, 2022). The first confirmed case of COVID-19 in Brazil was reported in December 2019 (Zhang T. et al., 2020). The latest name for the novel coronavirus is severe acute respiratory syndrome coronavirus 2 (SARS-CoV-2), which is a positive-sense single-stranded RNA virus (Di Gioacchino et al., 2021; Campbell et al., 2021). Phylogenetic analysis of the complete genome of the virus revealed that it was closely related to a group of previously discovered SARS-like coronaviruses (genus Betacoronavirus and subgenus Sarbecovirus) found in bats in China (nucleotide similarity of 89.1%). Currently, the genome is approximately 29.9 Kb in length and consists of 12 gene sequences (Figure 1) (Wu et al., 2020; Cresswell-Clay and Periwal, 2021; Gültekin and Allmer, 2021). One of the key factors in the epidemiological success of SARS-CoV-2 has been its ability to infect people who may remain asymptomatic for 14 days or more before the onset of disease symptoms. Therefore, early detection is very important (Oran and Topol, 2020). Currently, the most common methods used to detect SARS-CoV-2 include antibody and nucleic acid testing (Zhang J. et al., 2020; Chan et al., 2021; Trenti et al., 2021; Wang et al., 2021; Zhu et al., 2022). Laboratory parameters, severity, and clinical outcomes differ between antibody tests (Chen et al., 2021). Antibody testing has higher specificity for clinical applications to further diagnose individuals who have undergone preliminary confirmation; however, this method is more time-consuming and unsuitable for large-scale sample testing (Cardozo et al., 2020). Compared to antibody testing, nucleic acid testing is performed by collecting samples from the throat or respiratory tract of individuals and using techniques, such as polymerase chain reaction (PCR), to identify the presence of viral nucleic acids, thereby determining whether the individual is infected. This operational procedure is simple and suitable for testing large numbers of samples. In addition, virus gene testing has advantages over serum antibody testing in detecting infections earlier, with high specificity, early infection screening, and monitoring treatment effectiveness. It plays a very important role in epidemic monitoring, diagnosis, and treatment, especially in the control and intervention of early infection and virus transmission stages.

In order to improve the accuracy of nucleic acid detection, a more sensitive method called real-time fluorescent quantitative testing is often employed (Hussain et al., 2020; Liu et al., 2020). In conventional PCR testing, when the nucleic acid concentration in the sample is not sufficiently high, the amplification cycles may not be sufficient to detect the presence of the virus in patients. In contrast, the fluorescent quantitative PCR method is more practical for detecting low concentrations of the virus as it offers real-time monitoring, precision, higher sensitivity, and reduction of no-nspecific amplification effects, which is beneficial for the early detection of patients. To improve the quality of testing, probes are usually introduced in real-time fluorescence quantitative PCR (RT-qPCR). In RT-qPCR, the introduction of probes brings the advantages of high specificity, high accuracy, high sensitivity, and real-time monitoring, making it an important technology widely used in gene expression analysis, pathogen detection, gene quantification, and other fields (Haddar et al., 2020; Xiao et al., 2021). Currently, the most commonly tested gene sequences for the detection of the SARS-CoV-2 are ORF1a, BRAF, and N gene (Lee et al., 2021; Moitra et al., 2021; Varona et al., 2021). This method primarily relies on TaqMan RT-qPCR.

Influenza viruses are classified into three types: A, B, and C. Type A influenza viruses are further divided into numerous subtypes based on their H and N antigens (Le and Nguyen, 2014). Among them, subtypes H1N1, H2N2, and H3N2 are known to infect humans, while many other subtypes primarily circulate in various avian and animal species (Muñoz-Medina et al., 2015; Agustiningsih et al., 2023). Within avian influenza A virus (FluA), subtypes such as H1N1, H5N1, H7N1, H7N2, H7N3, H7N7, H7N9, H9N2, and H10N8 can cause acute respiratory infections in humans and animals (LI et al., 2013; Burke and Trock, 2018). Currently, the most susceptible and common upper respiratory tract infection influenza virus in humans is the influenza A subtype H1N1. Since 2009, it has caused outbreaks in Mexico, the United States, and other countries, rapidly spreading worldwide (Hale et al., 2010; Manicassamy et al., 2010). In just 1 year, it has killed more than 18,000 people in more than 214 countries and overseas territories or communities (Pandemic). The influenza A subtype H1N1 virus exhibits a phenomenon known as “reassortment,” where its genes come from the reassortment of four different subtypes of FluA (Libera et al., 2022; Luo et al., 2023). FluA is a negative-sense single-stranded RNA virus. Genome sequencing is approximately 13 kb in length (Lee, 2020; Tran et al., 2021). Currently, conventional PCR and RT-PCR are mainly used for the detection of upper respiratory tract infections caused by the influenza A subtype H1N1 virus. However, the low sensitivity of conventional PCR makes it less suitable for early clinical detection. According to the testing method published by the Chinese Center for Disease Control and Prevention (CDC) of the World Health Organization (WHO), multiple RT-PCR reactions are required, and there is a need for a larger number of labeled primer probes, which increases the cost and duration of the testing process (Agüero et al., 2007; Organization, 2009; Chander et al., 2010). Acute respiratory infections (ARI) kill nearly 4 million people each year. Viruses cause 30%–70% of ARI, with respiratory syncytial virus (RSV) and parainfluenza virus (PIV) accounting for the majority of these cases (Hatem et al., 2019). PIV is a common acute viral respiratory infection, primarily causing lower respiratory tract infections in infants and young children (Maeda et al., 2017; Zhang et al., 2021). In adults, it mainly manifests as an upper respiratory tract infections (Essa et al., 2017). PIV is a single-stranded negative-sense RNA virus with a genome length of approximately 15.6 kb, encoding 10 viral proteins (Ohkura et al., 2015; Hijano et al., 2018). Clinical diagnosis during the outbreak of PIV infection is relatively straightforward and often involves antibody and serum testing. However, during the early stages of infection, to detect low concentrations of the virus, fluorescent quantitative methods are still primarily used for TaqMan real-time fluorescent quantitative measurement of the conservative HN target gene of the virus (Chang-Ping et al., 2007; Xiu-Mei et al., 2014; Qi et al., 2023). RT-qPCR with a TaqMan probe was used to detect canine parainfluenza virus 5 (CPIV5) with high sensitivity and specificity, and can be used for detecting human parainfluenza virus type 3 (Jian-Fan et al., 2008; Jeon et al., 2023; Kim et al., 2023). RSV infection typically causes mild damage to the respiratory epithelial cells. However, in infants aged 2–6 months, it can lead to severe respiratory diseases, such as bronchiolitis and pneumonia. RSV can also cause upper respiratory tract infections, such as rhinitis and the common cold in older children and adults (Okabayashi et al., 2009; Percze et al., 2023). RSV is a negative-sense, single-stranded RNA virus. Its genome has a total length of 15.2 kb and encodes several proteins, including the F, G, and SH transmembrane proteins, M1 and M2 matrix proteins, and structural proteins such as N, P, L, NS1, and NS2 (Ma et al., 2021). Additionally, RSV can produce three unknown functional misc-RNA sequences. Currently, the method used for the rapid and accurate diagnosis of early human RSV infection still relies on TaqMan probe-based RT-qPCR detection. This method is more time-saving and labor-efficient than traditional virus isolation and culture, conventional PCR, nested PCR, and ELISA methods. Compared to conventional PCR and nested PCR, it requires a lower template concentration and has a sensitivity that is more than 10 times higher than that of the aforementioned methods. The target amplification gene used was the conserved N gene (Hu et al., 2003; AL-Bashar et al., 2017; You et al., 2017). At present, although the main detection method for the four viruses is RT-PCR, repeated screening is time-consuming and labor-intensive, and false positives caused by sampling may occur. Therefore, it is very necessary to establish a multiplex fluorescence quantitative PCR detection method for the detection of the SARS-CoV-2, FluA, PIV, and RSV.

Multiplex real-time fluorescence quantitative PCR (multiplex qPCR) is a technology that detects multiple genes in a single reaction by adding multiple pairs of specific primers and probes in a tube-based system; this is a rapid and sensitive method (Law et al., 2015). Zou et al. (2022) established a TaqMan multiplex qPCR for rapid detection of PCV2, PCV3, and PCV4. Yu et al. (2021) found that the TaqMan probe-based multiplex PCR assay can sensitively and specifically detect Ebola virus and Marburg virus simultaneously. This study aims to develop a one-step, fast, highly sensitive, and specific detection method based on multiple qPCR technology, and transform this method into a corresponding diagnostic kit to provide a fast and reliable method for a wider range of respiratory pathogen detection.

## 2 Materials and methods

### 2.1 Experimental project design and system establishment

#### 2.1.1 Experimental objective

To develop a one-step, rapid, highly sensitive, and specific detection method based on multiplex fluorescent quantitative PCR technology for respiratory pathogens, such as SARS-CoV-2, FluA, PIV, and RSV. The experimental scheme is shown in Figure 1. The aim was to transform this method into corresponding diagnostic reagent kits (Thermo Fisher Path-ID™ Multiplex One-Step RT-PCR Kit; Catalog number: 4442136).

#### 2.1.2 Experimental principles

This project introduces multiplex PCR to achieve simultaneous nucleic acid detection of multiple pathogens. The technical core of this method is the TaqMan probe real-time fluorescence quantitative PCR method. The basic principle is to use oligonucleotide probes that bind to the target sequence during the amplification process through the 5′ nuclease activity of the Taq enzyme. The probe is labeled with a fluorescent reporter group at the 5′ end and a fluorescence quencher group at the 3′ end, which is phosphorylated to prevent probe extension during the PCR process. When the primer extends to the binding position of the oligonucleotide, the Taq enzyme can cleave it into small fragments, separating the reporter group and the quencher group to emit fluorescence. Through the real-time detection of the fluorescence intensity increase during the amplification process, quantitative analysis of the samples is performed. Real-time detection of the fluorescence signal of each cycle of the PCR amplification reaction achieves quantitative and qualitative analysis of the initial template. The development of a rapid detection technology for multiple pathogens involves four steps: clarifying various pathogens, designing and synthesizing specific probes and primers, establishing standardized procedures, and developing reagent kits. In comparison to the longer detection cycle and more stringent experimental conditions of “*in vitro* culture” and the lower throughput quantitative PCR technology, this project introduces multiplex PCR technology to achieve one-step rapid simultaneous detection of various pathogens causing respiratory infectious diseases. It provides clinical differentiation diagnosis, guides antibiotic use, and assists clinical early, rapid, and accurate pathogen diagnosis. This is beneficial for early prevention and treatment of respiratory infectious diseases, precise medication, and timely and effective prevention and control of potential epidemics Table 1.

**TABLE 1:** Experimental instruments and consumables.

Instrument name	Manufacturer/Model
Fluorescent quantitative PCR instrument	Bio-rad (Bio-Rad CFX Connect)
Refrigerated benchtop centrifuge	Eppendorf 5810R
Mini handheld centrifuge	Eppendorf MiniSpin plus
Nucleic acid electrophoresis system	Beijing Liuyi DYCP-31DN
Gel imaging system	Shanghai Tianeng Tanon 1600
Vortex shaker	Shandong Boko QL-901
Nucleic acid concentration detector	Epoch biotek
Pipettes	Eppendorf
Various types of tips and centrifuge tubes	AXYGEN

#### 2.1.3 Experimental procedures

1. Experimental materials: SARS-CoV-2, FluA, PIV, and RSV were purchased from the National Health Commission Inspection Center with the appropriate permits. These standard strains were used for the experimental nucleic acid extraction.
2. Experimental instruments and consumables (Table 1):
3. Nucleic acid extraction: The nucleic acid samples of SARS-CoV-2, FluA, PIV, and RSV were extracted using the developed microvirus nucleic acid extraction reagent kit specifically designed for this project (all four viruses are RNA viruses). The extracted nucleic acid samples were quantified, adjusted in concentration, reverse-transcribed into cDNA, and stored at −80°C for later use.
4. Quantitative PCR sample preparation: The four sets of viral nucleic acid samples (cDNA) were grouped as follows: A single SARS-CoV-2 nucleic acid sample, B. Single FluA nucleic acid sample, C. Single PIV nucleic acid sample, and D. Single RSV nucleic acid sample. The initial concentration of the nucleic acid samples was carefully maintained to maintain consistency within each group.
5. Primer and probe design and synthesis: Based on the publicly available nucleic acid information of the four viruses, the specific primers and probes with different labels were designed using Primer Express 3.0 software.

The detection of SARS-CoV-2 involved the design of primers that specifically target the N gene (GenBank: OV016410.1).

S-CoV-2-F: AAC​TCC​AGG​CAG​CAG​TAG​GGG.

S-CoV-2-R: CAA​AGC​AAG​AGC​AGC​ATC​ACC.

The probe primers for detecting SARS-CoV-2 were as follows: S-CoV-2-t, CTC​CTG​CTA​GAA​TGG​CTG​GCA.

For the detection of FluA, primers targeting the M1 gene were designed (GenBank: MZ502953.1).

FluA-M1-F: CAC​AGA​AGT​GGC​GTT​TGG​C.

FluA-M1-R: AGC​GGG​TTG​GTG​GTG​GTT​A.

The probe primers for detecting FluA were FluA-M1-t and GTG​AGC​AGA​TTG​CTG​ATT​CAC​AGC​ATC​GAT.

For the detection of PIV, primers targeting the HN gene were used (GenBank: MH684390.1).

PIV-HN-F: CCA​GTT​ATG​CTC​CTT​GCC​CA.

PIV-HN-F: TCA​CAC​ACT​TGG​TGT​TGC​CT.

The probe primers for detecting PIV were as follows: PIV-HN-t: TAC​ACT​CGT​TTT​CCT​AGG​ATA​TGG​TG.

Primers targeting the detection of RSV, primers were designed for the N gene (GenBank: MG642050.1).

RSV-N-F: AGT​GAT​GTT​ACG​GTG​GGG​GGT.

RSV-N-R: ACT​TGT​TCC​ATT​TCT​GCT​TGC.

The probe primers for detecting RSV were as follows: RSV-N-t, CAG​TTA​AAA​ATA​TTA​TGT​TAG​GAC​ACG.

Reference primers (GenBank: AY582799.1) and their corresponding probes were designed [Biotree Biotech (Shanghai) Co., Ltd.].

β-actin F: CGG​GAC​CTG​ACT​GAC​TAC​CTC

β-actin-R: ATG​TCA​CGC​ACG​ATT​TCC​CGC.

The reference probe is as follows:

β-actin: CACCGAGCGCGGCTACAGCTTCACCACC.

6. The above five probe primers were entrusted to a company for different fluorescent labeling.

The S-CoV-2-t probe primer was labeled with ROX fluorescence, FluA-M1-t probe was labeled with CY5 fluorescence, PIV-HN-t probe was labeled with FAM fluorescence, and RSV-N-t probe was labeled with VIC fluorescence. The reference primer was labeled using TET fluorescence.

7. PCR Reaction System and Reaction Program

During the experiment, detection primers for the four viral nucleic acids were added to each group (A, B, C, D). Primer specificity was determined by simultaneously detecting the presence of different fluorescent signals. This indicates that different nucleic acid detection primers do not result in non-specific amplification between different species. Therefore, except for the reference primer, the remaining primer pairs were mixed and used for quantitative analysis with cDNA templates from the four groups.

The above-mentioned cDNA from groups A, B, C, and D were diluted 20-fold before the reaction. The reaction mixture was prepared in a 20 μL system. The specific configuration is as follows.

Premix Ex Taq 10 μL

ddH2O 5 μL.

F/R primer 1/1 μL (10 μM).

cDNA 3 μL.

The reaction program is as follows (40 cycles):

95°C 1 min.

95°C 10 s.

58°C 30 s.

65°C 10 s.

Using a Bio-Rad real-time fluorescence PCR instrument, fluorescence was collected at the detection wavelengths for ROX, CY5, FAM, VIC, and TET.

### 2.2 Detection limit test of multiplex PCR

To determine the detection limits of multiplex PCR assay for SARS-CoV-2, FluA, PIV, and RSV, we serially diluted RNA from the original samples by 10-fold to obtain 6 different concentration gradients. Plaque-forming unit (PFU) was used to measure the titer of SARS-CoV-2, and TCID50 method was used to measure the titers of FluA, PIV, and RSV.

### 2.3 Data analysis

GraphPad Prism 9.0 software was used for plotting. Comparisons between the two groups were performed using a *t*-test, and *p* < 0.05 was considered a significant difference.

## 3 Results

### 3.1 Related reagent kit development

In this project, a reagent kit was developed for the rapid, sensitive, and specific detection of respiratory pathogens, including SARS-CoV-2, FluA, PIV, and RSV. The reagent kit consisted of a sample nucleic acid extraction reagent and four pathogen-specific detection primers and probe powders. Quantitative reagents must be provided separately for use. The specific components of the reagent kit were DNA I 1000 U, Buffer R1 50 mL, Buffer R2 20 mL, Buffer W1 50 mL, Buffer W2 25 mL, RNase-free DEPC Water 20 mL, Spin columns with Collection Tubes 50 pcs, and RNase-Free Centrifuge Tubes (1.5 mL) 50 pcs.

Additionally, the reagent kit was accompanied by powdered primers and five probe powders, namely, S-CoV-2-F/S-CoV-2-R, FluA-M1-F/FluA-M1-R, PIV-HN-F/PIV-HN-R, RSV-N-F/RSV-N-R, and β-actin-F/β-actin-R. The primers and probes were stored at −20°C.

### 3.2 Reasonableness test of primer design

**FIGURE 2:**
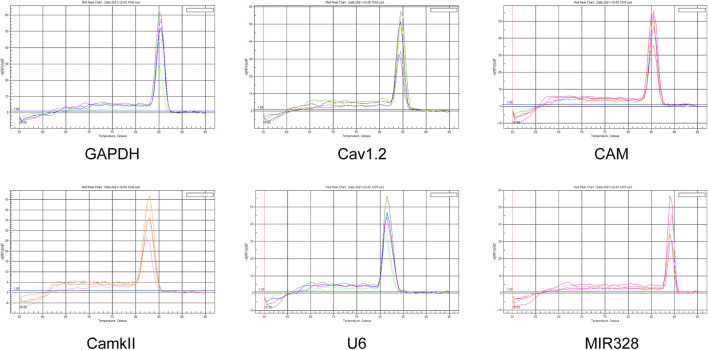
Baseline and threshold values for all primers.

Baseline refers to the background signal level before the primer was used in the experiment. The measurement of the baseline could help accurately identify the specific signals present in the experiment. The threshold was the critical point at which the signal was detected during PCR. When the signal exceeded a set threshold, we considered that a reliable amplification response had occurred, and that the signal could be confirmed as a positive result. Thresholds are often used to determine the presence or absence of amplification and assess the strength of amplification. Based on the combination of the primer baseline and threshold values in Figure 2, it can be judged that the target sequence was successfully amplified in the PCR experiment.

### 3.3 Multiplex fluorescent quantitative PCR detection profiles for respiratory pathogens

**FIGURE 3:**
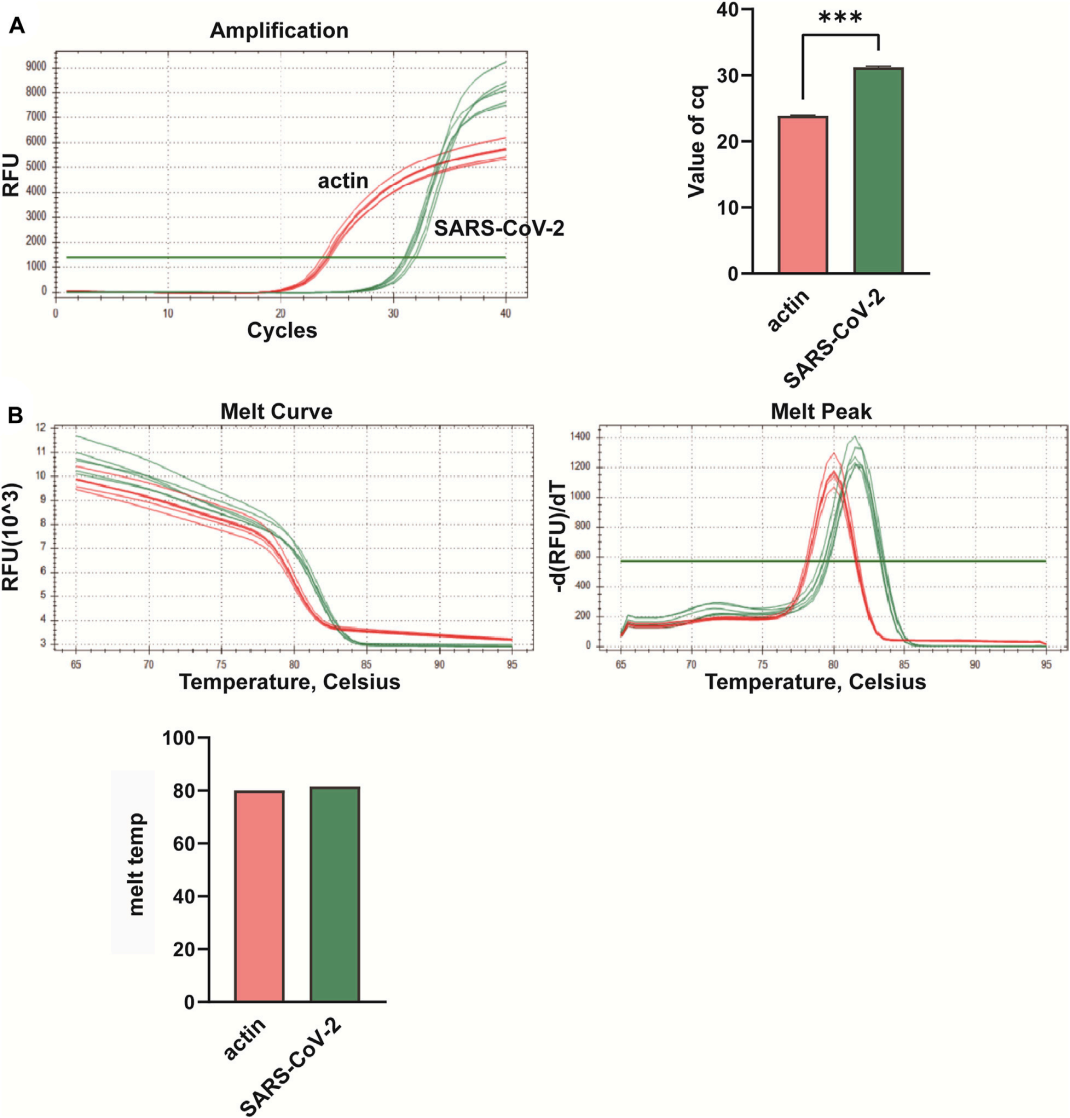
Multiplex fluorescent quantitative PCR detection profiles for Group A—Novel Coronavirus (SARS-CoV-2). **(A)** Cq values (cycle numbers at the threshold ct) of amplified samples; **(B)** Dissociation curves and melting temperatures of amplification curves. ***p < 0.001, *n* = 6.

**FIGURE 4:**
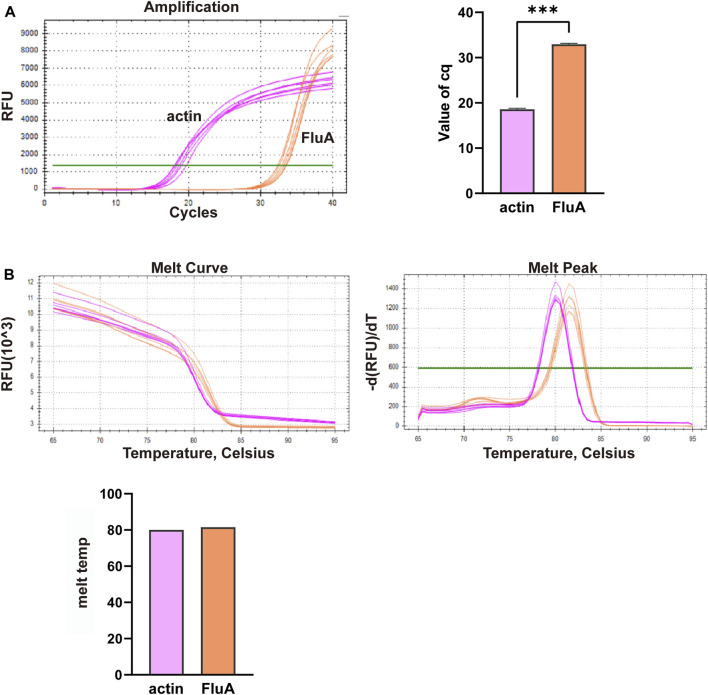
Multiplex fluorescent quantitative PCR detection profiles for Group B—Influenza A virus (FluA). **(A)** Cq values (cycle numbers at the threshold ct) of amplified samples; **(B)** Dissociation curves and melting temperatures of amplification curves. A total of 12 wells were run, including 6 replicates of actin samples and 6 replicates of FluA samples. ***p < 0.001, *n* = 6.

**FIGURE 5:**
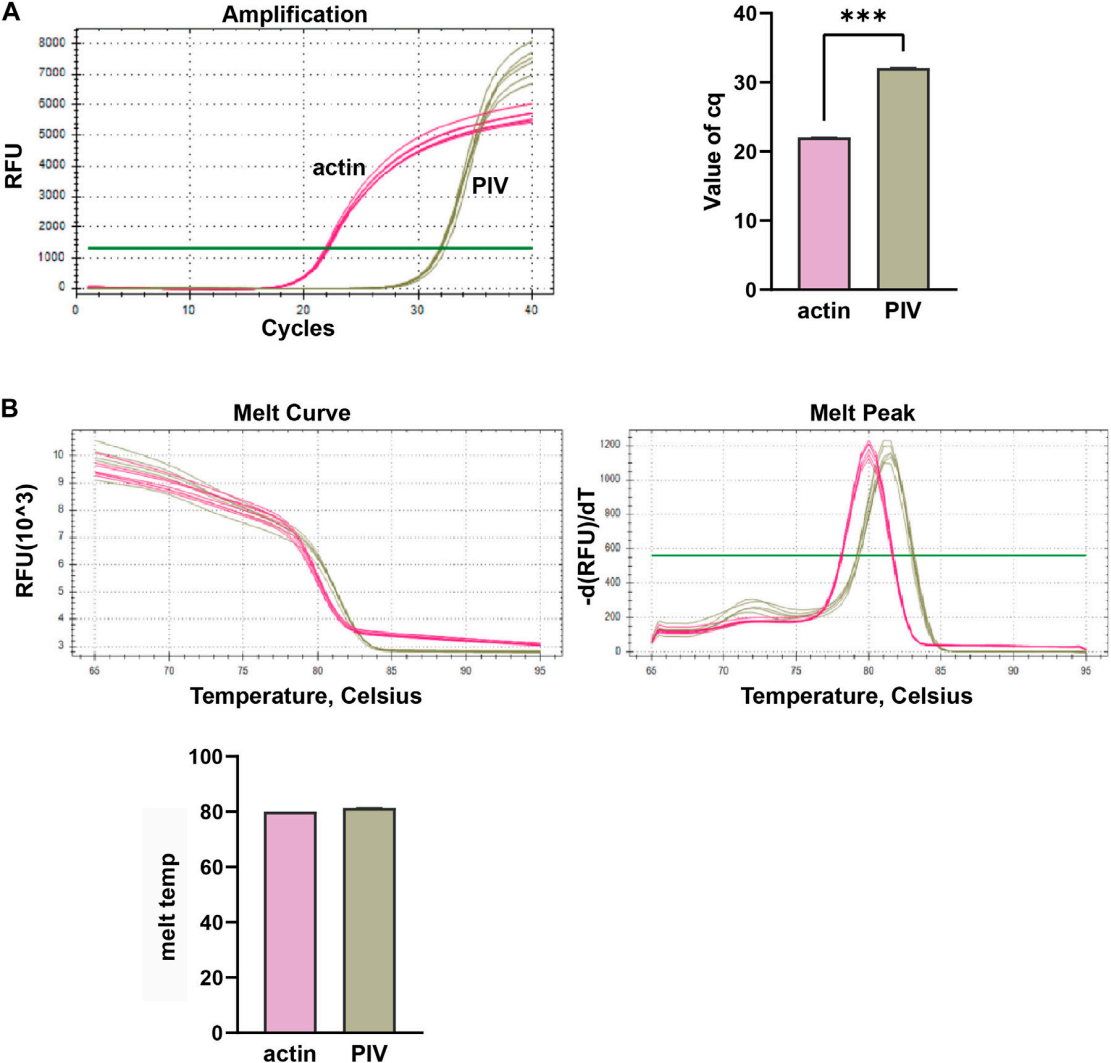
Multiplex fluorescent quantitative PCR detection profiles for Parainfluenza virus (PIV). **(A)** Cq values (cycle numbers at the threshold ct) of amplified samples; **(B)** Dissociation curves and melting temperatures of amplification curves. A total of 12 wells were run, including 6 replicates of actin samples and 6 replicates of PIV samples. ***p < 0.001, *n* = 6.

**FIGURE 6:**
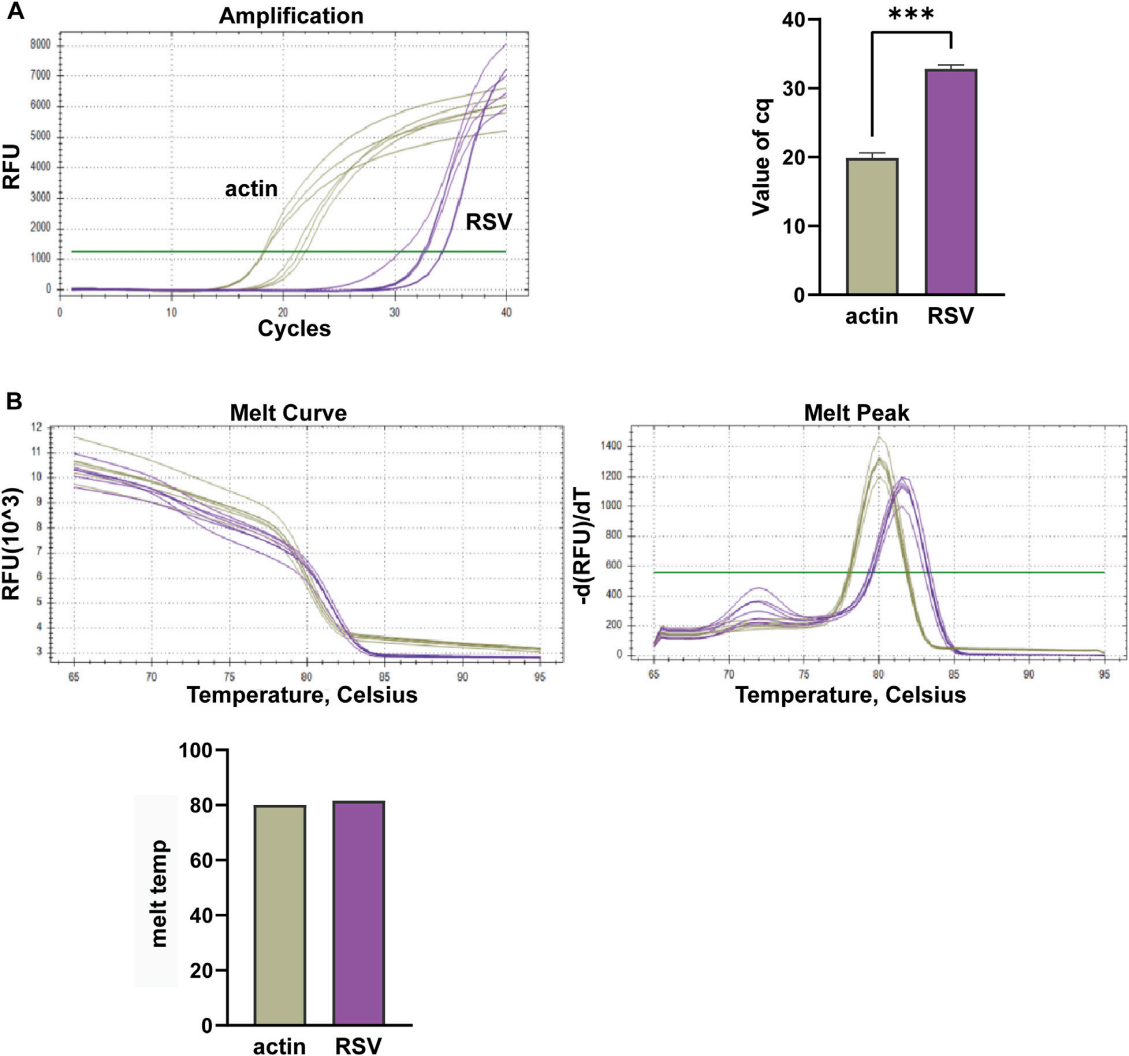
Multiplex fluorescent quantitative PCR detection profiles for Respiratory Syncytial Virus (RSV). **(A)** Cq values (cycle numbers at the threshold ct) of amplified samples; **(B)** Dissociation curves and melting temperatures of amplification curves. A total of 12 wells were run, including 6 replicates of actin samples and 6 replicates of RSV samples. ***p < 0.001, *n* = 6.

In this study, we conducted multiplex fluorescent quantitative PCR to analyze the presence of specific respiratory pathogens. Detection profiles were generated for the following pathogens: Group A, SARS-CoV-2; Group B, FluA; Group C, PIV; and Group D, RSV. Figure 3 shows the detection profile for Group A, focusing on the detection of SARS-CoV-2. We assessed the amplification samples for their Cq values, which represent the cycle number at the threshold Ct (Figure 3A). Additionally, the amplification curves were analyzed to obtain the dissociation curves and corresponding melting temperatures. The experiment included 12 wells with six replicates of actin samples and six replicates of SARS-CoV-2 samples (Figure 3B). Similar to the previous group, we determined the Cq values of FluA, PIV and RSV amplified samples, and obtained dissociation curves and melting temperatures from the amplification curves (Figures 4–6).

Overall, the detection profiles demonstrated specific fluorescence signals for the internal reference (actin) and the target nucleic acids in each reaction. From Figures 3–6, it can be seen that in each reaction (single nucleic acid sample), only two types of fluorescence signals for the reference and corresponding nucleic acid samples can be detected using five primers, and the peaks are single, indicating the absence of nonspecific amplification and demonstrating the strong specificity of the primers. The early peak times and generally smaller Cq values may suggest higher sensitivity of the assay, but they are not the only indicators used to determine sensitivity.

### 3.4 Detection limits of SARS-CoV-2, FluA, PIV, and RSV

The detection limits of SARS-CoV-2, FluA, PIV, and RSV were evaluated to assess the analytical performance of the assay kits (Table 2). The results indicate that the detection limit for SARS-CoV-2 is 1 × 10^−1^ PFU/mL, for FluA is 1 × 10^3^ TCID50/mL, for PIV is 1 × 10^1^ TCID50/mL, and for RSV is 1 × 10^1^ TCID50/mL.

**TABLE 2:** Limit of detection testing.

Virus	TCID50/mL &PFU/mL	SARS-CoV-2	FluA	PIV	RSV
		Cycle threshold values			
SARS-CoV-2	1 × 10^0^	26.11 ± 0.45	N/A	N/A	N/A
	1 × 10^−1^	30.13 ± 0.64	N/A	N/A	N/A
	1 × 10^−2^	N/A	N/A	N/A	N/A
	1 × 10^−3^	N/A	N/A	N/A	N/A
	1 × 10^−4^	N/A	N/A	N/A	N/A
	1 × 10^−5^	N/A	N/A	N/A	N/A
FluA	1 × 10^5^	N/A	24.36 ± 0.37	N/A	N/A
	1 × 10^4^	N/A	28.75 ± 0.42	N/A	N/A
	1 × 10^3^	N/A	33.43 ± 0.13	N/A	N/A
	1 × 10^2^	N/A	N/A	N/A	N/A
	1 × 10^1^	N/A	N/A	N/A	N/A
	1 × 10^0^	N/A	N/A	N/A	N/A
PIV	1 × 10^4^	N/A	N/A	21.83 ± 0.51	N/A
	1 × 10^3^	N/A	N/A	25.62 ± 0.24	N/A
	1 × 10^2^	N/A	N/A	29.51 ± 0.43	N/A
	1 × 10^1^	N/A	N/A	34.04 ± 0.39	N/A
	1 × 10^0^	N/A	N/A	N/A	N/A
	1 × 10^−1^	N/A	N/A	N/A	N/A
RSV	1 × 10^4^	N/A	N/A	N/A	21.98 ± 0.39
	1 × 10^3^	N/A	N/A	N/A	26.55 ± 0.31
	1 × 10^2^	N/A	N/A	N/A	31.59 ± 0.26
	1 × 10^1^	N/A	N/A	N/A	35.34 ± 0.15
	1 × 10^0^	N/A	N/A	N/A	N/A
	1 × 10^−1^	N/A	N/A	N/A	N/A

N/A: not available.

## 4 Discussion

In this study, we developed rapid nucleic acid testing techniques to detect common respiratory infectious diseases in the Chinese population. Multiplex PCR detection has several clear advantages. Compared with the single detection methods used in other studies, the multiplex PCR method showed higher specificity and sensitivity, allowing for the simultaneous detection of multiple pathogens in a single reaction. This approach not only saves time and resources, but also facilitates the rapid identification of various pathogens in complex samples (Liu et al., 2023). Our multiplex fluorescent quantitative PCR assays demonstrated high specificity, sensitivity, and efficiency in detecting respiratory pathogens, such as SARS-CoV-2, FluA, PIV, and RSV. The results of this study have important implications for clinical diagnosis, epidemiological surveillance, and public health intervention.

Multiplex PCR assays allow for the simultaneous detection of multiple pathogens in a single reaction, which significantly reduces testing time and cost (Lung et al., 2017; Liu et al., 2019). This is particularly valuable in the context of respiratory infectious diseases, in which timely and accurate diagnosis is crucial for effective disease management and control. By detecting multiple pathogens in a single assay, these techniques provide a comprehensive approach for respiratory pathogen screening, enabling healthcare providers to quickly identify the causative agents responsible for respiratory infections. Studies have shown that the multiplex qPCR assay developed allows for rapid screening of *Salmonella* spp., *S. Typhimurium*, and S. Enteritidis in various (food) matrices (Heymans et al., 2018). Therefore, this method can contribute to effective and rapid intervention. The application of multiple RT-qPCR in the early detection of bovine respiratory diseases in healthy calves revealed that multiple RT-qPCR can simultaneously analyze multiple pathogens, including viruses and bacteria, and contribute to the early detection of bovine respiratory diseases (Goto et al., 2023). One of the strengths of our developed technique is its high specificity, as demonstrated by the absence of non-specific amplification in the amplification curves. This indicated that the designed primer sets had strong specificity and minimized the risk of false-positive results. Specificity is crucial for accurate diagnosis because it ensures that the detected signals correspond to the targeted pathogens and not to other non-targeted genetic materials or contaminants.

Additionally, the high sensitivity of our assays, as indicated by their early peak times and relatively low Cq values, highlights their ability to detect low levels of target nucleic acids in clinical samples. This sensitivity is crucial for the early detection of infections, especially in cases in which the viral load may be low during the early stages of infection or in asymptomatic carriers. The ability to detect and identify pathogens with high sensitivity enables the prompt initiation of appropriate treatment, isolation, and containment measures. Researchers have found that each primer and probe set can only detect the target gene itself and cannot bind to any other target, indicating a high specificity. Based on bioinformatics analysis of each virus, multiple real-time RT-qPCR with primers and TaqMan probes targeting highly conserved regions of different virus genes was designed with high specificity and sensitivity for the simultaneous detection and differential diagnosis of porcine enteric coronavirus, PEDV, TGEV, PDCoV, and PEAV (Huang et al., 2019). The development and implementation of these rapid nucleic acid testing techniques has significant implications for public health in China. The ability to quickly and accurately detect respiratory pathogens allows for the timely identification of outbreaks, effective contact tracing, and implementation of targeted public health interventions. It also helps monitor the spread of infections, evaluate the effectiveness of control measures, and guide public health policies.

Despite the success of our technique, there are several limitations that should be acknowledged. First, the assays were validated using a limited number of clinical samples. Further validation studies with larger sample sizes are necessary to establish their robustness and generalizability. Additionally, assays were developed specifically for the targeted respiratory pathogens mentioned in this study, and their applicability to other respiratory pathogens should be further investigated.

In conclusion, our study demonstrated the successful development and validation of rapid nucleic acid testing techniques for the detection of common respiratory infectious diseases in the Chinese population. These techniques offer a sensitive, specific, and efficient approach to simultaneously detect multiple pathogens and provide valuable tools for clinical diagnosis, epidemiological surveillance, and public health interventions. Further research and validation are required to expand the scope of these techniques and ensure their broader applicability in clinical practice and public health settings.

## 5 Conclusion

In this study, we successfully developed rapid nucleic acid testing techniques for common respiratory infectious diseases in a Chinese population. Our multiplex PCR assays demonstrated high specificity and sensitivity for detecting respiratory pathogens, including SARS-CoV-2, FluA, PIV, and RSV. These techniques offer a rapid and reliable method for the simultaneous detection of multiple pathogens, thereby saving time and resources. Further research and validation are needed to optimize and expand the application of these techniques to a broader range of respiratory pathogens and to enhance their utility in clinical and public health settings.

## Data Availability

The original contributions presented in the study are included in the article/Supplementary material, further inquiries can be directed to the corresponding authors.
